# Dynamical patterns underlying response properties of cortical circuits

**DOI:** 10.1098/rsif.2017.0960

**Published:** 2018-03-28

**Authors:** Adam Keane, James A. Henderson, Pulin Gong

**Affiliations:** 1 School of Physics, The University of Sydney, New South Wales 2006, Australia; 2 ARC Centre of Excellence for Integrative Brain Function, The University of Sydney, New South Wales 2006, Australia; 3 Cancer Council NSW, Sydney, New South Wales 2011, Australia

**Keywords:** cortical circuits, neural response properties, balanced excitation and inhibition, asynchronous state, neural variability

## Abstract

Recent experimental studies show cortical circuit responses to external stimuli display varied dynamical properties. These include stimulus strength-dependent population response patterns, a shift from synchronous to asynchronous states and a decline in neural variability. To elucidate the mechanisms underlying these response properties and explore how they are mechanistically related, we develop a neural circuit model that incorporates two essential features widely observed in the cerebral cortex. The first feature is a balance between excitatory and inhibitory inputs to individual neurons; the second feature is distance-dependent connectivity. We show that applying a weak external stimulus to the model evokes a wave pattern propagating along lateral connections, but a strong external stimulus triggers a localized pattern; these stimulus strength-dependent population response patterns are quantitatively comparable with those measured in experimental studies. We identify network mechanisms underlying this population response, and demonstrate that the dynamics of population-level response patterns can explain a range of prominent features in neural responses, including changes to the dynamics of neurons' membrane potentials and synaptic inputs that characterize the shift of cortical states, and the stimulus-evoked decline in neuron response variability. Our study provides a unified population activity pattern-based view of diverse cortical response properties, thus shedding new insights into cortical processing.

## 1. Introduction

Understanding how cortical circuits respond to sensory stimulation is of fundamental importance in elucidating the mechanisms of cortical processing [1]. Extracellular recordings have shown that trial-to-trial rate variability declines after stimulus onset, whereas spike time variability is retained [2]. Recent whole-cell recordings have also revealed that sensory stimulation can shift cortical neurons from synchronous to asynchronous states, as characterized by the dynamics of membrane potentials [3,4]. Seemingly unrelated to these response properties measured at the level of individual neurons, it has been found that there exist distinct spatio-temporal patterns in neural population response activity, depending on the strength of feed-forward thalamic input signals [5]. In one response pattern, a stimulus with low contrast triggers a wave that propagates across cortical circuits; in another response pattern, a stimulus with high contrast evokes a neural response that remains spatially localized and does not propagate to neighbouring areas. To deepen our understanding of cortical processing, it is important to unravel the mechanistic links between these response properties of cortical circuits across different levels, and account for them in a unified way.

Randomly coupled networks with balanced excitation and inhibition (E/I balance) are the standard model used to account for spike time variability; in these networks, the firing rates of excitatory and inhibitory neurons adjust dynamically, resulting in an asynchronous state in which different neurons emit spikes in an irregular and asynchronous way [6,7]. To account for the variability of firing rates and its decline, as observed in [2], balanced networks have been extended to incorporate clustered connections [8]. Consistent with these studies, various experimental studies have shown that in cortical circuits, excitation is often balanced by inhibition [9–12]. However, these existing studies of cortical networks with balanced E/I do not capture the infrequent, large excursions of membrane potential observed during spontaneous activity [4]. Furthermore, explaining the shift from synchronous to asynchronous states and stimulus strength-dependent population response patterns in cortical circuits with E/I balance, remains an open question.

In this study, we unravel the dynamical mechanisms of the essential response properties by exploring a qualitatively different model of balanced cortical circuits, which incorporates the widely observed distance-dependence of synaptic connectivity [13,14]. We show that in this spatially extended network, population-level response patterns are dependent on the strength of external stimuli: weak stimuli evoke propagating waves but strong stimuli evoke localized activity patterns without propagation. These stimulus strength-dependent population response patterns are quantitatively comparable with those reported in [5].

We illustrate that propagating waves during spontaneous activity or weak stimulation, when passing a neuron, produce transient, synchronized synaptic inputs to the neuron, and can thus account for infrequent yet large fluctuations of membrane potential with non-Gaussian dynamics, as observed in the synchronous state [4,15]. In this state, neural spiking dynamics in our circuit model have both variability of spike timing and slow fluctuations of firing rates, as found in [2]. However, localized activity patterns evoked by strong stimuli have irregular spiking configurations, generating synaptic inputs with nearly Gaussian dynamics to individual neurons. These uncorrelated inputs cause neurons to be continuously depolarized, emitting spikes in an asynchronous way, but without firing rate fluctuations. The changes in the dynamics of the population-level response patterns evoked by strong stimuli can, therefore, explain the shift from the synchronous to the asynchronous states [4] and the decline in trial-to-trial firing rate variability [2]. Our study thus unravels the dynamic mechanism underlying the cortical response properties, significantly advancing our understanding of cortical processing.

## 2. Material and methods

### 2.1. Spiking circuit model

We consider a two-dimensional network of *N* × *N* coupled, conductance-based leaky integrate-and-fire (LIF) neurons (*N* = 300 in this study). We denote the membrane potential of a neuron at integer coordinates **r** = (*x*, *y*) and time *t* as *V*_**r**_(*t*), whose dynamics are described by2.1
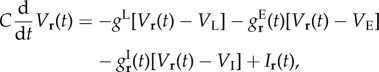
where *g*_L_ = 25 nS is the leak conductance, *C* = 0.5 nF is the capacitance, and *V*_L_ = − 70 mV, *V*_E_ = 0 m and *V*_I_ = − 80 mV are the leak, excitatory and inhibitory reversal potentials, respectively. The network consists of 75% excitatory and 25% inhibitory neurons, arranged in an evenly spaced lattice, with the inhibitory neurons at gridpoints where both *x* and *y* are odd. If *V*_**r**_(*t*) reaches the spike threshold *V*_th_ = − 55 mV, the neuron at **r** generates a spike and its membrane potential resets to *V*_R_ = − 70 mV for a refractory period *τ*_ref_ = 5 ms [16]. The synaptic conductances are denoted by *g*^*ζ*^_**r**_(*t*) where *ζ* = *E*, *I* indicates excitatory and inhibitory conductances, respectively. Their dynamics are described by2.2
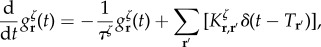
where *T*_**r′**_ is the time of the spikes emitted by the afferent neuron located at ***r*′** = (*x*′, *y*′), *δ*_**r**_(*t*) is the Dirac delta function, and *τ*^E^ = 2 ms and *τ*^I^ = 2 ms are the characteristic decay times for excitatory and inhibitory conductances, respectively. Spiking excitatory neurons contribute to *g*^E^_**r**_(*t*) and spiking inhibitory neurons contribute to *g*^I^_**r**_(*t*).

The average distance between a neuron and its nearest neighbour is approximately 30–40 μm [17,18]. For consistency, we assume a distance of 40 μm. Both excitatory and inhibitory connections are constrained to 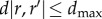, where 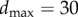 neurons = 1200 μm, the same order of magnitude as long-range connections [19]. Using this maximum range, each neuron in our model receives 2820 afferent connections.

The coupling strength *K*^*ζ*^_**r**, **r′**_ between afferent pairs of neurons located at **r** = (*x*, *y*) and **r′** = (*x*′, *y*′) is described by2.3
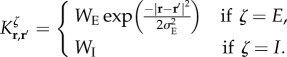
The Gaussian profile for *K*^E^_**r**, ***r*′**_, with amplitude *W*_E_ = 7.5 × 10^−3^ nS and the spatial decay *σ*_E_ is a first approximation to the empirical evidence that coupling strengths or connection probabilities between neurons decrease as distances between them increase [13,14]. We use 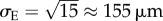, in agreement with experimental values of pyramidal-to-pyramidal projections [13]. The homogeneous inhibitory coupling strength *W*_I_ = 5.0 × 10^−3^ nS matches anatomical evidence that inhibitory connections to pyramidal neurons are non-specific and dense [20]. There are no substantial consequences insofar as one could certainly obtain similar observations with an inhibition of sufficient strength that decays sufficiently slowly as a function of distance (see electronic supplementary material, figure S1); the choice of a uniform inhibition between afferent neurons (i.e. up to 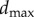) also allows us to better characterize a change in balance by adjusting the inhibition; in this case, we do not need to account for the effects of the shape of the connection strength.

The uniform inhibition between afferent neurons in our model results in the range of inhibitory connections being greater than that of excitatory connections. There is evidence supporting this; for example, in layer 3 of macaque cortex (visual areas V1, V2 and V4; somatosensory areas 3b, 1, and 2; motor area 4; and prefrontal cortical areas 9 and 46), basal dendrites of pyramidal cells have 200 μm diameter, whereas the five long branches of basket cells have a 650 μm radius (Fig. 3 in [21]). It has also been found that in basket cells of cat area 18, local (i.e. less than 1 mm) inhibition extends further than excitation [22]. On the other hand, from a functional standpoint, it is known that the centre-on and surround-off connectivity reflects such an arrangement of long-range lateral inhibition and short-range excitation. It has been found that in V1 of macaque monkeys, such cortical surround suppression can only be modelled as a result of a larger inhibitory spatial range compared to excitation [23]. By studying auditory and visual stimuli in V1 of owls, the authors pointed out why other studies, which use equal ranges of E/I, cannot match key aspects of their data [23]. Another study showed that, in addition to classical inhibitory surrounds, there exists global inhibition, which has several different properties [24]; for example, inputs from the classical inhibitory surround interact additively with inputs from a unit's excitatory centre, which help to shape the tuning of neurons to nearby stimuli. By contrast, inputs from the global inhibitory surround interact divisively with inputs outside the receptive field [24]. These interactions enable distant stimuli to suppress neuronal responses to stimuli within the receptive field [24]. It has also been pointed out that in addition to broad inhibition from large basket calls, local disinhibition from cells such as double-bouquet cells, may contribute to a Mexican-hat-like arrangement [25]. Our network is a first approximation to such a functional arrangement.

The external excitatory input, included in equation (2.1), is given by2.4
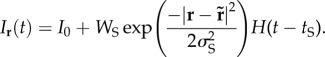
For times of *t* < 2 s, all neurons receive a background stimulus of *I*_0_ = 0.4 nA. However, after *t*_S_ = 2 s, an additional localized stimulus is added, as indicated by the Heaviside function *H*( · ). This localized stimulus is centred around a certain gridpoint 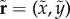 in the network; we term the circular area (of radius 15 gridpoints) near this centre the region of input (RoI). We use a spatial Gaussian profile of amplitude *W*_S_ and spatial decay 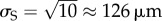 to approximate cortical receptive fields.

To run the model, we use the Euler method with time step d*t* = 0.05 ms, but similar results can be obtained for smaller values of d*t*. The initial membrane potentials are chosen from a random uniform distribution with values ranging between *V*_R_ =− 70 mV and *V*_th_ =− 55 mV. We use a lattice of size *N* = 300 with periodic boundary conditions, in order to avoid finite-size effects. To study the response property of the network model, we perform an analysis on an 81 × 81 section of this grid centred around the RoI, except where otherwise noted. We perform 500 trials for a given set of conditions, and each trial is run for 3.5 s, with the first 1 s excluded. The code for running the model with this parameter set is available at https://github.com/BrainDynamicsUSYD/spikegrid.

## 3. Results

### 3.1. Stimulus strength-dependent population response patterns

**Figure 1.**
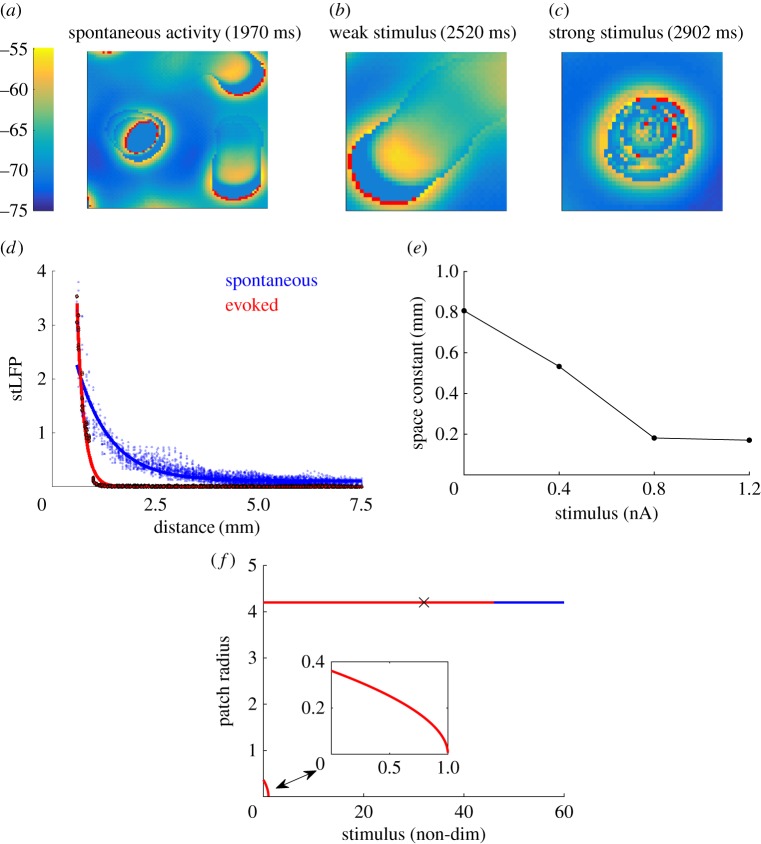
Properties of spontaneous and evoked activity patterns. (*a*–*c*) Colours in the images represent membrane potential values between −75 mV (blue) and spike threshold *V*_th_ = − 55 mV (yellow), as indicated by the colour bar, with red pixels indicating neurons that have fired within the last millisecond. Each image is of a network of size 300 × 300 neurons, but a smaller range is shown for clarity, namely an 81 × 81 subsection in (*a*) and a 41 × 41 subsection centred around the region of input (RoI) in (*b*) and (*c*). (*a*) Snapshot of the spatio-temporal patterns emerging from the balanced, spatially extended network during the spontaneous activity. These patterns take the form of multiple, localized patchy patterns and crescent-shaped propagating waves. (*b*) Snapshot of a propagating wave evoked by a weak stimulus (0.4 nA) within the RoI. (*c*) Snapshot of a localized patchy pattern evoked by a strong stimulus (1.2 nA) within the RoI. (*d*) Spike-triggered local field potential (stLFP) as a function of distance from the RoI for the spontaneous activity (blue dots) and activity evoked by a strong stimulus (black dots). The blue line and red line are exponential fits to the data for the spontaneous and evoked activity, respectively. (*e*) The average propagation range, as characterized by the space constant, of the response patterns varies as a function of stimulus strength. (*f*) Stability of two localized solutions of the firing rate model as a function of stimulus strength. The inset shows a zoomed in version of the solution with smaller radius. Red indicates values that are unstable to perturbation, while blue indicates stable values. The ‘x’ indicates the point (approx. 34) at which numerical simulations show a shift from the case in which perturbations cause propagating waves to the case in which the localized patchy pattern remains stationary.

We consider a spatially extended, conductance-based spiking circuit model with excitatory and inhibitory neurons (see Material and methods). This network model incorporates the distance-dependent coupling property that has been found in the connections of cortical neurons at different levels [13,14]. Another key property of the network is that its E/I are approximately balanced; that is, the ratio of excitatory and inhibitory synaptic inputs to individual neurons is around 1. At the population level, the balanced, spatially extended network exhibits spontaneous activity in the form of propagating wave patterns with complex dynamics (figure 1*a*). This balance is robust across a range of inhibitory strengths, namely 2.5 × 10^−3^ ≤ *W*_I_ ≤ 7.5 × 10^−3^ nS (see Material and methods); for *W*_I_ ≥ 7.5 × 10^−3^ nS, the patterns tend to be static, or wander around a restricted area; and for *W*_I_ ≤ 2.5 × 10^−3^ nS, the patterns are not isolated from one another, but expand and merge. This study is built around the observation that these patterns can capture the complexity of propagating waves as found during spontaneous cortical activity [26–32]. The dynamics of these patterns can, in turn, quantitatively account for a range of experimental observations of the irregular dynamics of spontaneous neural activity [28].

In this study, we address the fundamental problems regarding the intrinsic network mechanism underlying stimulus strength-dependent response patterns [5], the shift from the synchronous to the asynchronous state [3,4], and the decline in neural variability caused by sensory stimuli [2]. In addition, we unravel the mechanistic relationship between these essential neural response properties. For these purposes, we add deterministic external stimulation to a localized region of the circuit (equation (2.4)), which we refer to as the RoI. This local input is based on the consideration that cortical inputs are topographically organized; for instance, in the visual cortex, thalamic inputs are organized such that neighbouring cortical neurons represent adjacent portions of the visual field [1].

For the spatially extended network, the RoI generally exhibits two distinct spatio-temporal response patterns, namely localized patchy patterns and propagating waves; their relative occurrence is a function of stimulus strength. Figure 1*b* shows a propagating wave evoked by applying a weak stimulus to the RoI. The dynamics of these evoked waves are similar to those of the propagating waves that occur during the spontaneous activity, with random, seemingly superdiffusive, long-range trajectories (electronic supplementary material, figure S2a). These waves consist of a crescent-shaped spiking front with a refractory wake; similarly shaped waves have been observed, for example, in rat visual cortex [33]. Behind the refractory wake is a ‘ball’ of depolarized neurons (figure 1*a*,*b*). This is likely due to the spiking wavefront and background stimulus *I*_0_ (see Material and methods) providing excitation to non-refractory neurons that are also largely outside of the strong inhibitory fields of other patterns. A strong stimulus, however, would evoke a localized patchy pattern that is confined to the RoI (figure 1*c*), and wanders around this zone with seemingly Brownian motion (electronic supplementary material, figure S2b); outside the RoI, the network exhibits spontaneous dynamics as shown in figure 1*a*. If the strong stimulus is removed, the RoI rapidly returns to the dynamics of the spontaneous network. These results are consistent with the empirical observation that cortical responses exhibited two distinct spatio-temporal modes (i.e. propagating waves and localized patchy patterns), which were generated when visual stimulus was weak (or absent) and strong, respectively [5].

The average propagation speeds of the underlying spiking patterns emerging from the RoI decrease when a strong stimulus is applied. The spontaneous speed is approximately 44 mm s^−1^, similar to that found experimentally in rat visual cortex [33], whereas the evoked speed is approximately 25 mm s^−1^, which reflects the trapping of many of the evoked patterns. However, to quantify how the propagation range of the population response patterns changes as a function of stimulus strength, as in [5], we must calculate the local field potential (LFP) for our spiking circuit model. To do this, we use a method based on synaptic currents to obtain a temporal LFP component and convolve it with a spatial LFP component [34], which consists of a Gaussian envelope with width comparable to the typical extent of LFP [35] (see the electronic supplementary material). As in [5], we then calculate the spike-triggered local field potential (stLFP), that is, the peak amplitude of the LFP induced by an evoked spiking pattern. In our model, this stLFP is calculated at evenly spaced intervals of 5 gridpoints, corresponding to a cortical distance of 200 μm, but similar results can be obtained for modest changes in how it is calculated (see the electronic supplementary material). Note, that a similar result could be found in any part of the grid when no stimulus is applied; in this case, the stLFP is independent of the RoI. However, by comparing the RoI before and after the stimulus, we can use it as a ‘control’ to show how the dynamics of patterns emerging from the same zone alter once a stimulus is applied. A comparison between the patterns emerging from each zone of the grid is performed in Nauhaus *et al.* [5]; in our case, however, we are effectively just concentrating on one zone.

In our model, the stLFP decays as a function of distance from the RoI during both the spontaneous and evoked activity (figure 1*d*, blue and black dots). As in [5], this decay can be fit as an exponential function *M* exp(−d/λ) + *B*, with a space constant λ (figure 1*d*, blue and red lines). The space constants for the spontaneous activity and the activity evoked by a strong stimulus are λ_spont_ = 0.8 mm and λ_evoked_ = 0.175 mm, respectively. This result quantifies the observation that a strong stimulus evokes responses that are more spatially localized than spontaneous activity patterns, which occur in the absence of stimuli. The space constant ratio (i.e. λ_spont_/λ_evoked_) in our model is 4.7 ± 0.2, which is quantitatively comparable with those reported in [5], namely a value of 5.5 ± 2.1 for cats, and 3.0 ± 2.3 for monkeys.

Such a dramatic change in the spatial profile of stLFP (figure 1*d*) may represent the endpoint of a gradual change caused by progressively stronger stimuli. To test this, we calculate the stLFP at intermediate values of stimulus strength. We find that the modulation of external stimuli causes the propagation range, as characterized by the space constant, to decrease linearly up to a stimulus strength of 0.8 nA (figure 1*e*). This linear decrease occurs because localized patchy patterns, which tend to be confined to the RoI, become more common as the stimulus strength is increased. On the other hand, the crescent-shaped waves, which usually leave the RoI rapidly, become less common. Consequently, the average distance travelled by evoked patterns becomes smaller. For large enough stimulus strengths, only localized, persistent patchy patterns are evoked, meaning that the average range of propagation is limited only to the RoI, where these patterns are confined. These patchy patterns are sustained indefinitely until the stimulus is removed. This corresponds to the levelling out effect that occurs when stimulus strengths are greater than 0.8 nA (figure 1*e*); further increases in stimulus strength do not significantly alter the propagation range.

#### 3.1.1. Theoretical analysis of stimulus strength-dependent response patterns

To obtain a further understanding about the mechanism underlying the stimulus strength-dependent response patterns, we consider a firing rate model that approximates the spiking circuit model, and is analytically tractable (see electronic supplementary material). To preserve E/I balance, the firing rate model has the same ratio of excitatory to inhibitory inputs as that in the spiking circuit model (see electronic supplementary material, equation S8). The spontaneous activity of the firing rate model exhibits propagating wave patterns with complex dynamics, resembling the patterns emerging from the spiking circuit model (figure 1*a*). Similarly, for the firing rate model, a weak stimulus tends to evoke a propagating wave, but a strong one evokes a localized patchy pattern.

To understand why different population response patterns are evoked by weak (or absent) and strong stimuli, we construct an explicit localized solution to the firing rate model and analyse its stability under different eigenmode perturbations (see the electronic supplementary material). When stimuli with different strengths are added to the firing rate model, we find that it has two solutions (figure 1*f*): one solution with a smaller radius and another one with a larger radius. For the firing rate model, the radius of the localized solution with the large radius stays almost the same as the stimulus strength increases; this property is similar to that of the evoked localized pattern in the spiking circuit model. Our analysis shows that the solution with the smaller radius is unstable for all stimulus strengths (figure 1*f*, lower red line). However, based on our stability analysis, we find that the localized solution with the larger radius is stable to perturbations when stimulus strength is greater than or equal to 47 (figure 1*f*, blue line), and unstable when stimulus strength is less than or equal to 46 (figure 1*f*, upper red line). For the unstable case, small perturbations would cause a localized patchy pattern to evolve into a propagating wave. Numerical simulations of the firing rate model confirm that this change in stability largely coincides with a change in pattern dynamics: for weaker stimuli, a localized patch solution subjected to a small perturbation can propagate away as a wave, but for stronger stimuli, it remains stationary (figure 1*f*, cross).

### 3.2. Stimulus-evoked shift from the synchronous to asynchronous states

Another fundamental property of cortical responses is that stimuli shift neural activity from the synchronous to the asynchronous state, as observed in [3,4]. We now demonstrate that in our model, a strong stimulus can shift the state of the RoI from the synchronous to the asynchronous state, and that the resultant changes in membrane potential dynamics are comparable to those directly measured by using intracellular, whole-cell recordings [3,4]. In addition, we illustrate that the different population activity patterns occurring during the spontaneous and evoked activity provide a mechanistic account of the temporal properties of synaptic inputs to individual neurons that accompany the different cortical states.

#### 3.2.1. Membrane potential dynamics

**Figure 2.**
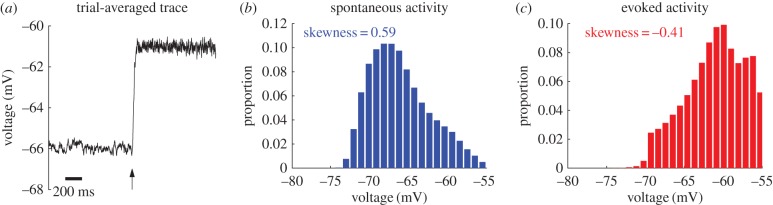
Membrane potential properties related to the synchronous and asynchronous states. (*a*) Membrane potential of a randomly chosen neuron within the region of input (RoI) averaged across all trials; the arrow indicates the time at which the stimulus is turned on, which coincides with a substantial rise in the mean value towards spike threshold (*V*_th_ = − 55 mV). (*b*) Histogram of the membrane potential values of the randomly chosen neuron during the spontaneous activity, aggregated across all trials; it has a positive skewness value of 0.59. (*c*) Histogram of the membrane potential values of the randomly chosen neuron within the RoI after stimulation, aggregated across all trials; it has a negative skewness value of −0.41.

Whole-cell membrane potential measurements from the primary visual cortex of behaving monkeys show that in the synchronous state, the average membrane potential during spontaneous activity is generally far from the spike threshold [4]. However, in the asynchronous state that occurs after stimulus onset, measurements show that the membrane potential approaches this threshold [4]. This is the case for the membrane potential of individual neurons in our model: the time series of membrane potential for a randomly chosen neuron, averaged over all trials, exhibits a rapid ascent to a larger value after a strong stimulus is applied (figure 2*a*). To quantify this observation, we calculate the distance, averaged over time, between its membrane potential and the spike threshold (i.e. 〈*V*〉 − *V*_th_). For the neuron shown in figure 2*a*, the median value of 〈*V*〉 − *V*_th_ across all trials is 11.0 mV for the spontaneous activity, which is similar to the value of 13.9 mV reported in [4]. However, after stimulus onset, this value changes to 6.1 mV, confirming that membrane potential is closer to the spike threshold, which is a characteristic feature of the asynchronous state [4]. Applying this analysis to the entire RoI, we obtain a median value of 〈*V*〉 − *V*_th_, averaged across neurons, of 10.9 mV prior to stimulation, and 7.6 mV after stimulation.

Another characteristic change in the dynamics of membrane potential, which accompanies a shift from the synchronous to the asynchronous state, is related to its distribution. In the synchronous state, the large, occasional excursions in membrane potential result in a non-Gaussian distribution with a heavy tail at depolarized potentials [4]. In our model, as shown in figure 2*b*, the histogram of membrane potential for a randomly chosen neuron during the spontaneous activity has such a non-Gaussian distribution. We can quantify how heavy-tailed a unimodal distribution is by calculating its skewness *S*. Skewness measures the symmetry of a distribution with respect to its median: *S* > 0 indicates that the mean is greater than (to the right of) the median, whereas *S* < 0 indicates that the mean is less than (to the left of) the median (see the electronic supplementary material). We find that across all trials, the distributions of the randomly chosen neuron have a median skewness of *S* = 0.59, meaning that there is a heavy tail at depolarized values of its membrane potential. If we apply this analysis to all neurons within the RoI, we find that the average of these median skewnesses is 0.59 (s.d. 0.02), similar to the median skewness of 0.72 reported in [4]. This positive skewness value indicates that during the spontaneous activity there are large, infrequent excursions in membrane potential [4]. Such non-Gaussian distributions of membrane potential have also been measured in the auditory cortex of both awake and anaesthetized rats during spontaneous activity [15,36].

In the asynchronous state, many uncorrelated inputs cause the distribution of membrane potential to be approximately Gaussian or to have a slightly negative skewness [4]. The histogram for a randomly chosen neuron in our network after stimulus onset is consistent with this (figure 2*c*): across all trials, the membrane potential distributions of this neuron have a median skewness of *S* = − 0.41. Note that the depolarized tail of this histogram is truncated as a byproduct of the integrate-and-fire neural model, which can only capture subthreshold values of membrane potential, that is, values less than −55 mV (see Material and methods). However, if we apply this analysis to all neurons within the RoI, we find that the average of these median skewnesses is −0.09 (s.d. 0.38); this is within the margin of error of the value of *S* = 0 expected for a Gaussian distribution, and is consistent with whole-cell recordings of evoked activity [4].

#### 3.2.2. Synaptic input dynamics

**Figure 3.**
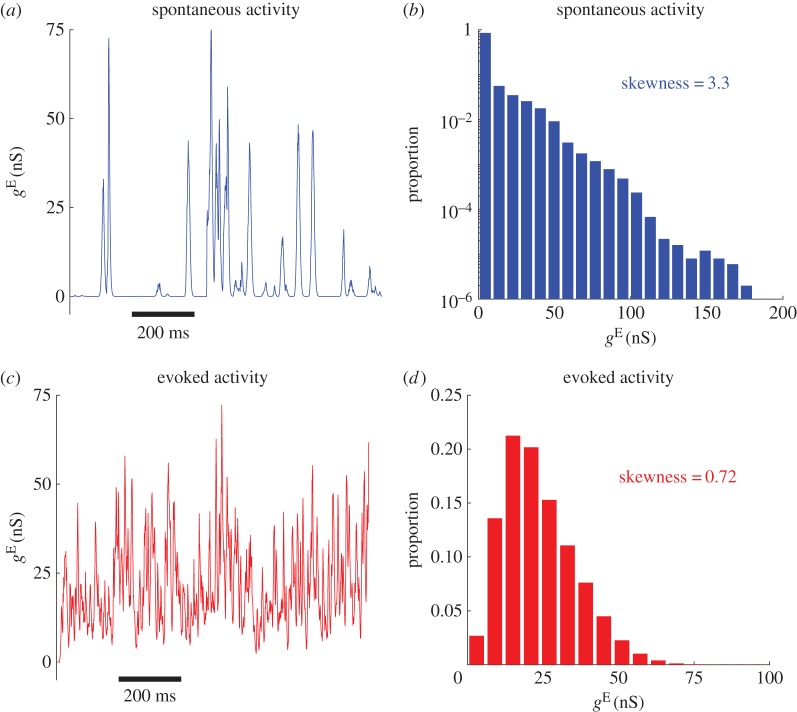
Properties of synaptic inputs to neurons during the spontaneous and evoked activity. (*a*) Time series of the excitatory conductance *g*^E^ received by a randomly chosen neuron within the RoI during the spontaneous activity; it consists of quiescent activity punctuated by large, occasional excursions in conductance. (*b*) Histogram of the excitatory conductance *g*^E^ received by the neuron during the spontaneous activity, aggregated across all trials; it is heavy-tailed, as emphasized by the use of logarithmic scaling for the *y*-axis, with a skewness value of 3.3. (*c*) Time series of the excitatory conductance *g*^E^ received by the neuron after stimulation; it consists of continuous fluctuations about a mean. (*d*) Histogram of the excitatory conductance *g*^E^ received by the neuron across all trials after stimulation; it more closely resembles a Gaussian, with a skewness value of 0.72.

We now illustrate that the membrane potential dynamics occurring during the different cortical states are synaptic in origin. In our spatially extended network, the excitatory synaptic conductance *g*^E^ to individual neurons during the spontaneous activity consists of quiescent periods punctuated by large, transient excursions, indicating the arrival of transient synchronized inputs to the neuron (figure 3*a*). The resultant histogram of *g*^E^ received by this neuron has a long tail (figure 3*b*), indicating that the synaptic inputs which add to *g*^E^ have non-Gaussian dynamics. To further quantify these non-Gaussian dynamics, we again calculate the skewness, and find that the median skewness of the distribution is 3.3, which is significantly larger than 0. Note that such heavy-tailed distributions of synaptic inputs have been observed in brain slices [37] and somatosensory cortex [38]. This is in agreement with previous findings in an unstimulated, spatially extended network with balanced E/I [28].

After the onset of stimulus, however, *g*^E^ has fundamentally different dynamics: it now fluctuates continuously, with smaller amplitudes than during the spontaneous activity (figure 3*c*). The distribution of *g*^E^ has a median skewness of 0.72 (figure 3*d*), which is significantly smaller than that found during the spontaneous activity; in other words, the dynamics more closely resemble that of a Gaussian distribution. This change in skewness occurs across the RoI, with an average median of skewnesses of 3.32 (s.d. 0.02) during the spontaneous activity, and 0.81 (s.d. 0.59) after stimulus onset. These results thus demonstrate that in the synchronous state during the spontaneous activity, synaptic inputs to individual neurons are transiently synchronized. These synchronized events are randomly distributed in time (figure 3*a*) while the inputs have non-Gaussian dynamics. By contrast, in the asynchronous state evoked by strong stimuli, these inputs have more Gaussian dynamics.

#### 3.2.3. Population response patterns underlie the different synaptic dynamics

**Figure 4.**
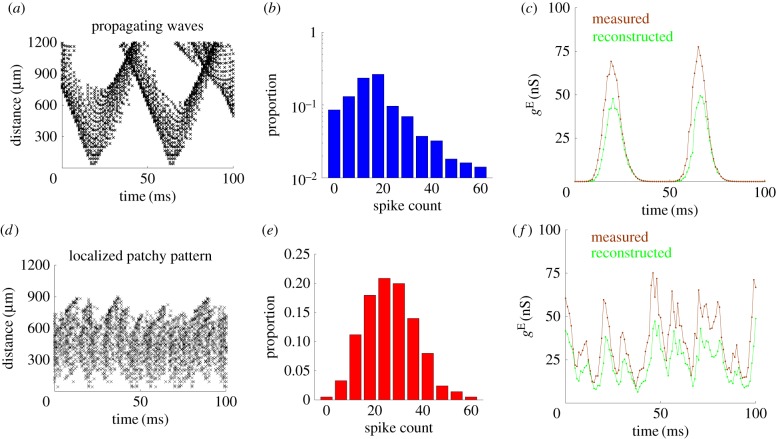
Population response patterns underlie the shift from the synchronous to the asynchronous state. (*a*) Average distance from spiking neurons, with each point depicted by a ‘ × ’, in propagating wave fronts to a test neuron during the spontaneous activity. The average distance rapidly decreases as a wave approaches and rapidly increases as a wave recedes. (*b*) Histogram of the number of spikes received by a test neuron, calculated using a Δ*t* = 1 ms sliding window, for one trial during the spontaneous activity. Note the logarithmic scaling of the *y*-axis, emphasizing that the distribution is heavy-tailed. (*c*) The time series of excitatory conductance *g*^E^ received by the test neuron during the spontaneous activity (brown line) closely matches the time series based on the spiking data (green line) in figure 4*a*. (*d*) Average distance from spiking neurons in a localized patchy pattern, each depicted by a ‘ × ’, to a test neuron in the RoI with a strong stimulus. Because the spikes occur at seemingly irregular configurations within the pattern, there is a constant fluctuation in the distances at which they occur. (*e*) Histogram of the number of spikes received by a test neuron, calculated using a Δ*t* = 1 ms sliding window, for one trial during the activity evoked by a strong stimulus. The histogram appears to be approximately Gaussian. (*f*) The time series of excitatory conductance *g*^E^ received by the test neuron during the activity evoked by a strong stimulus (brown line) closely matches the time series based on the spiking data (green line) in figure 4*d*.

We next illustrate that the dynamics of the population-level response patterns, as we have demonstrated above, provide an explanation for the temporal properties of synaptic inputs. During the spontaneous activity, a wavefront provides a source of synchronized input to any neurons that it approaches. We can quantify this dynamic mechanism by considering the distance between a randomly chosen ‘test’ neuron within the RoI and the spikes occurring in a wavefront that is approaching it. The wavefront consists of multiple spikes occurring within a short time interval (figure 4*a*); as soon as the distance from the wavefront to the test neuron is smaller than *D*^E^ = 1200 μm (see Material and methods), the spiking neurons in the wave front are afferent to the test neuron and thus provide synchronized input to it. However, the rapid movement of the propagating wave means that after it has passed by, it quickly recedes such that the distance again exceeds *D*^E^. After this, the spiking neurons in its wavefront no longer provide inputs to the test neuron; such synchronous inputs are, therefore, transient, resulting in the bumpy features of the time series of *g*^E^ received by the test neuron (figure 3*a*).

We can quantify the number of spikes that the test neuron receives from the propagating wave as a function of time by using a sliding window of duration Δ*t* = 1 ms, a short duration that is necessary to capture the transient, synchronized dynamics of the synaptic inputs. The resultant histogram of spike counts, calculated across a 1000 ms interval, has a heavy tail (figure 4*b*). This indicates that the test neuron usually receives few spikes except when a wave passes by, which provides the large spike counts contributing to the heavy tail. By comparing the distances of the spikes (figure 4*a*) with the time series of *g*^E^ received by the test neuron (figure 4*c*, brown line), we can see that the large excursions (bumps) in input do indeed occur at the same time as and when the spikes in each wavefront are closest.

As a final illustration of the relationship between the wavefront and the synaptic inputs, we reconstruct the time series of *g*^E^ received by the test neuron, based on the spike distances shown in figure 4*a*. First, for each millisecond time interval, we calculate the amount that each presynaptic spike contributes to *g*^E^ according to equation (2.3). We then add each of these contributions to the *g*^E^ received by the test neuron, which is evolved according to equation (2.2). The resultant time series shows that, based on the propagating wavefront, we are able to reproduce the bumpy synaptic dynamics (figure 4*c*, green line).

When a strong stimulus is applied, a localized patchy pattern is usually evoked; this pattern is confined to the RoI. At each moment, the pattern has a seemingly irregular spiking configuration, which generates a sustained fluctuation in the distances at which spikes occur, as shown in figure 4*d*; the average of these distances over time is significantly less than *D*^E^. The resultant histogram of the number of afferent spikes that the test neuron receives in a Δ*t* = 1 ms interval is bell-shaped (figure 4*e*); this indicates that it receives nearly Gaussian synaptic inputs. By comparing the distances of the spikes (figure 4*d*) with the time series of *g*^E^ received by the test neuron, we can see that the random changes in the spiking configuration of the localized patchy pattern do correspond to fluctuations in *g*^E^ (figure 4*f*, brown line). By applying the same method described in the previous paragraph to the spikes shown in figure 4*d*, we are again able to reconstruct a time series of *g*^E^ that is similar to the measured one (figure 4*f*, green line).

These results, therefore, demonstrate that the dynamics of the synaptic inputs to the neurons in the RoI can be explained by the dynamics of the population response patterns. The synchronized synaptic inputs caused by propagating waves have non-Gaussian dynamics (figures 3*b* and 4*b*), resulting in large, infrequent excursions of membrane potential as measured for the synchronized state [4]. The localized patchy patterns generate synaptic inputs with more Gaussian dynamics (figures 3*d* and 4*e*), and the resultant membrane potential close to the spike threshold; this indicates that the state of neurons in the RoI with the strong external stimulus is the asynchronous, balanced state [4].

### 3.3. Stimulus-evoked decline in neural firing variability

**Figure 5.**
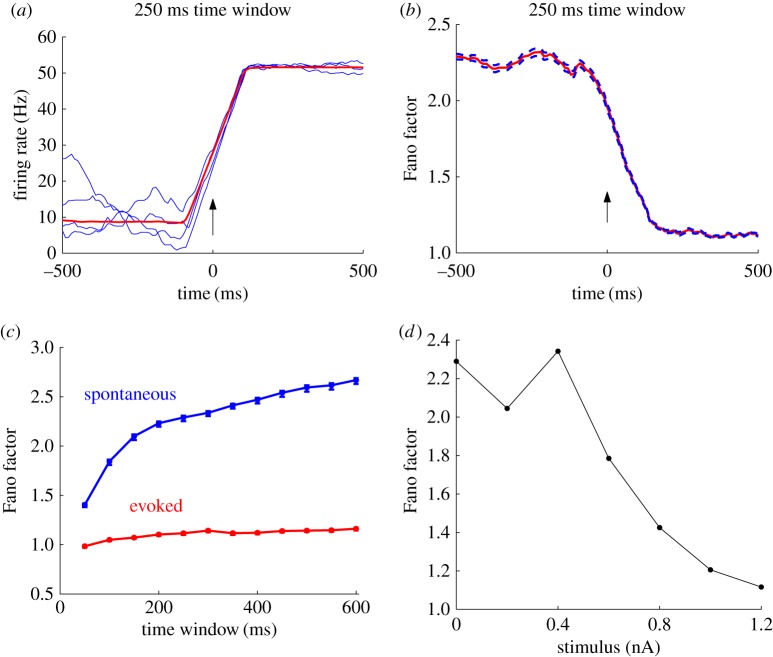
Stimulus onset quenches neural variability. (*a*) The firing rate, calculated using a Δ*t* = 250 ms sliding time window, averaged over all neurons within the RoI for five individual trials (blue lines) and averaged over all 500 trials as well as all neurons within the RoI (red line). The arrow indicates the stimulus onset time; after the stimulus is switched on, the trial-averaged firing rate increases and individual trials fluctuate much less. (*b*) Fano factor (red solid line with flanking blue dashed lines denoting standard error), which is calculated using a Δ*t* = 250 ms sliding time window and averaged over all neurons within the RoI. The arrow indicates the stimulus onset time; after the stimulus is switched on, there is a large decrease in Fano factor value. (*c*) Fano factor as a function of time window Δ*t* during the spontaneous activity (blue line) and after stimulation (red line). For Δ*t*≥150 ms during the spontaneous activity, Fano factor increases linearly with time window; after the stimulus is applied, the Fano factor remains the same value regardless of the choice of Δ*t*. (*d*) Fano factor, calculated using a Δ*t* = 250 ms sliding time window and averaged over all neurons within the RoI, as a function of stimulus strength; for larger stimulus strengths, there is a monotonic decrease in Fano factor.

We now demonstrate that our spatially extended network with E/I balance is able to explain the stimulus-evoked decline in neural variability that has been widely observed in the cortex [2], and show that this prominent feature of cortical responses can be explained by the population-level response patterns. To this end, we first calculate the spike counts across all trials of the neurons within the RoI by using a sliding window of size Δ*t* = 250 ms. During the spontaneous activity, the average firing rate is approximately 9 Hz (figure 5*a*, red line), which is the same order of magnitude as that found in experimental studies [39]. However, there are temporal and trial-to-trial fluctuations in firing rates across all neurons in the RoI (figure 5*a*, blue lines); these fluctuations are due to the occasional dynamic switching of individual neurons between the periods of high firing and low firing rates, similar to that observed in previous modelling studies [8,28].

#### 3.3.1. Fano factor indicates quenching of trial-to-trial variability

To quantify the changes in neural variability in our model, we calculate the mean-matched Fano factor (FF), as in [2] (see the electronic supplementary material). The use of mean-matching ensures that any changes in FF are not trivially related to changes in firing rates. We note, however, that even without mean-matching, the FF exhibits the same qualitative effects. During the spontaneous activity, the FF of each neuron is roughly the same across the network, and has a value of 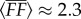 (figure 5*b*) where 〈 · 〉 indicates averaging over time and the bar indicates averaging over neurons within the RoI; such FF values that are well above 1 have been widely observed experimentally during spontaneous cortical activity [2,40,41]. Because a Poisson point process without any variations in its underlying rate has an FF of 1, values in excess of this indicate that there are fluctuations in firing rates in addition to irregular spike timing; neural variability can thus be approximated as a doubly stochastic process [42].

After stimulus onset, there is a sharp increase in firing rate, a significant reduction of firing rate fluctuations within and across trials (figure 5*a*), and a sharp decline in the FF to 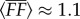 (figure 5*b*). These results are quantitatively consistent with experimental observations; for instance, in macaque monkeys, the neural variability assessed by the FF using the same time window that we use (Δ*t* = 250 ms), has been reported to decline from an initial value of approximately 2.1 to a final value of approximately 1.5 in MT, and from an initial value of 2.3 to a final value of 1.3 in dorsal premotor cortex ([2], their electronic supplementary material, figure S3). In our model, if the stimulus is switched off again, both the firing rate and FF of neurons within the RoI return to the original values of the spontaneous activity; this process is also in agreement with experimental observations in cortical areas, such as in V1 and MT ([2], their electronic supplementary material, figure S4).

As pointed out above, the FF can reflect variability due to both the irregular timing of individual spikes and fluctuations in firing rate. Therefore, to unravel which of these factors is largely responsible for the decline in FF, we calculate the FF as a function of the size of the time window Δ*t* within which spikes in the RoI are counted. During the spontaneous activity, the FF increases as a function of time window for Δ*t* ≤ 600 ms, and for Δ*t* ≥ 150 ms this increase is approximately linear (figure 5*c*, blue line); for Δ*t* > 600 ms, the FF levels out, becoming less sensitive to the size of the time window. The linear dependence of FF on the time window for 150 ≤ Δ*t* ≤ 600 ms indicates that individual neurons exhibit fluctuations in firing rates over these timescales; this is because a larger time window captures more of the switches between the high and low firing rate states that happen over these timescales. However, for the activity evoked by the strong stimulus, the FF does not depend on the size of the time window (figure 5*c*, red line); this indicates that there are no transitions between high and low firing rate states because each individual neuron is in the high firing state. Nonetheless, the FF value is still 1.1, which is similar to that expected for a Poisson process without fluctuations in firing rates. In other words, after stimulus onset, the variability of precise spike timing remains but the trial-to-trial fluctuations of spike rates decline, as found in [2]. This evoked activity, therefore, cannot be approximated as doubly stochastic but is consistent with the classical balanced state [43]. Note, however, that this conclusion only applies to the mean-matched values (see the electronic supplementary material).

#### 3.3.2. Population response patterns account for the decline in trial-to-trial rate variability

The disappearance of doubly stochastic firing activity, and the resultant decline in FF values at stimulus onset, can be explained in terms of the population response patterns. During the spontaneous activity, the rapid movement of propagating waves provides a brief source of synaptic input as their spiking wavefront sweeps past a particular neuron (figure 4*a*). Their random origins and trajectories, therefore, cause the variability of spike times over short timescales. On the other hand, the sustained activity of localized patchy patterns (figure 4*d*) causes the high firing state. Because these patterns also have random origins and trajectories, they cause stochastic switching to the high or low firing rate states as they wander towards or away from individual neurons, respectively. As mentioned previously, this dynamic switching provides the basis for firing rate fluctuations over long timescales. Thus, the coexistence of both kinds of activity patterns is the mechanism underlying doubly stochastic firing activity, as elucidated further for spontaneous cortical activity in [28].

However, as shown in figure 1*e*, response patterns have a smaller propagation range when they are evoked by strong stimuli. This is because for strong stimuli, the RoI is constantly occupied by a localized patchy pattern, causing the high firing state to occur for most of the neurons within the RoI. Because the localized patchy pattern is confined to the RoI and cannot move away, there is, therefore, no switching to low firing rate states. Thus, while there is still irregularity in spike times over short timescales due to the seemingly irregular spike configurations within the localized patchy pattern (figure 4*d*), there are no firing rate fluctuations over long timescales. This is evidenced by the Gaussian distribution that a random number of spikes with a constant rate would be expected to produce (figure 4*e*). It should also be noted that neurons in the periphery of the RoI receive a large amount of inhibitory input due to the centre-surround inhibition of the localized patchy pattern, and are consequently quiescent, rather than having a high firing state. Nonetheless, due to the immobility of the evoked, localized patchy pattern, these neurons do not exhibit any switching behaviour either, instead remaining in the quiescent state.

#### 3.3.3. Trial-to-trial rate variability depends on applied stimulus strength

We now go beyond the previous findings of a decline in neural variability caused by stimulus onset [2], to investigate how neural variability, as measured by FF, changes as a function of stimulus strength (figure 5*d*). While the value of FF fluctuates for weak stimuli (less than or equal to 0.4 nA), for stronger stimuli (greater than or equal to 0.4 nA) there is a monotonic decline. This decline in neural variability can also be understood in terms of the population response patterns. As shown in figure 1*e*, the average propagation range of response patterns decreases linearly as a function of stimulus strength up to strong values (greater than or equal to 0.8 nA) where it levels out. As pointed out earlier, this linear decrease occurs because localized patchy patterns, which tend to be confined to the RoI, become more common as the stimulus strength is increased. The increasing probability of localized patchy patterns being evoked means that individual neurons in the RoI are more likely to be in the high firing state. Furthermore, because these localized patchy patterns can be confined to the RoI, individual neurons are less likely to switch to the low firing rate state, which occurs when a localized patchy pattern moves away from the RoI. This means that there would be progressively less fluctuations in firing rate, because most trials would only exhibit the high firing state associated with localized patchy patterns and not the low firing rate state associated with propagating waves. However, figure 5*d* also shows that for weaker stimulus values (0.2–0.4 nA), there can be a slight increase in FF as a function of stimulus strength. To understand this, we note that during the spontaneous activity, the occurrence of sustained localized patchy patterns is rare, and thus there are only occasional transitions to the high firing rate state, which quickly return to the low firing rate state. A small stimulus can cause a more equal ratio between the occurrences of localized patchy patterns and propagating waves, thereby maximizing the number of switches between their associated high and low firing rate states. This, in turn, causes a larger degree of trial-to-trial variability as measured by FF.

## 4. Discussion

In this study, we have shown that dynamical response properties at different neural levels, as found in recent experimental studies, are mechanistically related in spatially extended, spiking neural circuits with balanced E/I. Population response patterns in these circuits are dependent on the strength of external stimuli, as found in [5]. As we have illustrated, these response patterns, which include propagating waves and localized activity patterns, can account for the stimulus-evoked change from the synchronous to the asynchronous state [3,4], and the decline in trial-to-trial rate variability [2]. This mechanism, while explaining the changes in stLFP, is caused by the underlying spiking patterns of individual neurons, as elucidated in figure 4, and thus provides an explanation rather than just an emulation for these observations. Whereas these previous models may be able to explain one of these stimulus-based characteristics (e.g. [8] can explain the reduction in trial-to-trial variability), our model is able to explain all of these observations in a unified way. Our results thus unravel the dynamic mechanism underlying these experimental observations, which otherwise remain disjointed in the existing cortical network models with balanced E/I [6–8,44].

Cortical responses during spontaneous activity and weak sensory stimulation generally take the form of propagating waves spreading along lateral connections [5,45]. A strong focal stimulus, however, tends to evoke localized responses restricted to the input region. This finding of stimulus strength-dependent response patterns has been proposed to reconcile two apparently opposing views of cortical processing [5], namely the view that the responses of cortical neurons are largely determined by local processing of thalamic inputs [46,47], and the view that cortical responses are substantially shaped by lateral connections [48,49]. By quantitatively reproducing the characteristic features of the stimulus strength-dependent response patterns found in [5], our network model provides mechanistic plausibility for reconciling the two views of cortical processing as proposed in [5]. In addition, our results demonstrate that such stimulus strength-dependent response patterns are an emergent property of the spatially extended network with balanced E/I. Our results thus indicate that different response patterns can coexist in a single balanced network. Consequently, there is no need to introduce different neural mechanisms for stimuli with different strengths, such as disynaptic inhibitory signals for strong stimuli as proposed in [5], to account for the stimulus strength-dependent response patterns.

Population response patterns emerging in our spatially extended network can account for a variety of seemingly unrelated, prominent response features of individual neurons. Propagating wave patterns with complex dynamics arising from the balanced network produce synchronized, bumpy synaptic inputs that are randomly distributed in time for individual neurons; the magnitudes of these inputs are heterogeneous, with a heavy-tailed distribution (figure 3*b*). These synchronized inputs cause non-Gaussian membrane potential fluctuations, consisting of quiescent periods that are occasionally interrupted by short intervals of high amplitude depolarization. Such synchronized synaptic inputs, and the resultant non-Gaussian membrane potential dynamics, have been widely observed in whole-cell recordings [3,4,15,36,50,51]; the accompanying cortical state is generally referred to as the synchronous state [4]. Here, we emphasize that the synchronous state, as studied here and found in experimental studies, is a transient synchrony, during which individual neurons receive synchronized synaptic inputs from presynaptic neurons within a short period of time; this is different from the stable synchrony addressed in previous modelling studies [52], during which all neurons fire synchronously all the time. The transient synchrony, as illustrated above, is caused by the localized wave pattern sweeping through a local area of the network. This is different from global transient synchrony, i.e. the synchronous irregular state found in sparsely, randomly coupled networks [53].

Aside from accounting for the synchronous state during spontaneous activity, our results unravel the dynamic mechanism underlying the shift from the synchronous to asynchronous states evoked by strong stimulation, as found in whole-cell membrane potential measurements from the cortex of behaving monkeys [4]. As we have demonstrated, when a strong external stimulus is present, a localized patchy pattern would be trapped in the RoI. In this case, there would not be any wave patterns sweeping through the RoI, because it would already be occupied by the localized patchy pattern; this means that no transient, synchronized inputs, like those found during the spontaneous activity, can be formed. However, the localized, evoked patchy pattern produces nearly Gaussian synaptic inputs to the neurons within it, because it has irregular spiking configurations that are not correlated over time. Accordingly, the cortical state with such random synaptic inputs is consistent with the asynchronous state, in which different neurons in the RoI asynchronously emit spikes, and their membrane potentials are closer to the spike threshold than those during the spontaneous activity. Local bump activity patterns pinned by a local external input have been studied in firing rate models, but these rate patterns cannot capture irregular spiking configurations within the localized spiking pattern in our model [54–58]; these irregular spikes, however, are crucial for explaining experimental data, as demonstrated in our study. Propagating waves can be stabilized to obtain stationary bumps by applying a constant input with a spatially localized Gaussian profile [59], and the formation of spatio-temporal patterns has been studied in spatially extended spiking networks with excitation and inhibition. However, in these studies, the collective dynamics of population activity patterns have not been mechanistically related to the experimentally observed response propertie as illustrated here in our study.

The decline in trial-to-trial firing rate variability has been modelled in balanced networks with clustered connectivity [8]. In [8], the mechanics underlying the fluctuations of firing rates is spontaneous switching between two attractors, with one attractor representing a low-activity state and the other one representing a high-activity state. Stimuli bias networks towards the high-activity state, thus reducing firing rate fluctuations. However, as we have demonstrated in our model, the change of the collective dynamics of propagating patterns at the population level causes the decline in firing rate variability. The balanced cortical networks with random connectivity have been highly successful at explaining the asynchronous and irregular nature of spike timing of cortical neurons [6,7]. Very recently, it has been found that variable neural activity with certain spatial correlation structures can emerge from a spatially extended, balanced cortical circuit when driven by external stimuli [44]. Nevertheless, these previous cortical models cannot explain the other essential response properties of cortical circuits, including: the dynamics of membrane potential with large, infrequent fluctuations during spontaneous activity [4,36]; the shift from the synchronous to asynchronous state [4]; and the stimulus strength-dependent population response patterns [5]. These important empirical observations can, however, be captured by our spatially extended network model with emergent, dynamical patterns, including the propagating wave patterns and localized patchy patterns. Our results thus provide a new and unified, dynamical pattern-based framework to understand cortical processing.

Propagating wave patterns at the circuit level have indeed been widely observed in the cortex [26,27,29–32,45,60]. In particular, high-density neural recordings have begun revealing concurrent propagating wave patterns like those found in our model. For instance, based on whole brain recordings of transparent fish, multiple propagating patterns, which are termed as a spatial gradient of activity timing, have been observed [61]. In [30], the two types of activity patterns, namely localized patchy patterns and propagating waves, have been explicitly documented. To test the mechanistic relationships between the essential neural response properties, as unravelled in our study, it would be ideal to combine imaging studies and massive multi-unit recordings to visualize and record neural activity at different levels, and to analyse emergent population response patterns with different stimulation strengths. This could be done in conjunction with the analysis of membrane potential, synaptic input and neural variability by using the same methods as we have done in our modelling study. This would allow us to validate the relationship between post-synaptic potentials and the average distance to each spiking pattern in the neuron, as predicted in figure 4. In addition, our work predicts that (figure 5*d*) for intermediate stimulus values, the FF is a nonlinear function of stimulus strength. This could be verified by measuring the FF *in vivo* at multiple stimulus strengths.

## Supplementary Material

Supplementary Materials for Dynamical Patterns Underlying Response Properties of Cortical Circuits

## Data accessibility

The code for running the model with this parameter set is available at: https://github.com/BrainDynamicsUSYD/spikegrid.
