# Supplementary material for: Dynamical patterns underlying response properties of cortical circuits
Source: J R Soc Interface. 2018 Mar 28;15(140):20170960. doi: 10.1098/rsif.2017.0960 (PMC5908533; doi:10.1098/rsif.2017.0960)
Supplement: Supplementary Materials for Dynamical Patterns Underlying Response Properties of Cortical Circuits [file rsif20170960supp1.pdf]

Supplementary Materials

Dynamical Patterns Underlying Response Properties of Cortical Circuits

Adam Keane, James Henderson, Pulin Gong

Journal of the Royal Society Interface

Methods

Spike-triggered local field potential

In [11], the propagation range of neural responses is quantified by the spike-triggered local field potential (stLFP). To quantitatively compare the response properties of our model with those characterised by stLFP in [11], we first calculate the local field potential (LFP). Previous models have demonstrated that a linear combination of the absolute values of excitatory and inhibitory currents can act as a first approximation to the temporal component of the LFP [10]. For a neuron positioned at $\mathbf{r} = (x, y)$, the equation for this approximation is:

$$LFP_{\text{time}}(\mathbf{r}, t) = |g^E_{\mathbf{r}}(t)(V_{\mathbf{r}}(t) - V_E)| + |g^I_{\mathbf{r}}(t)(V_{\mathbf{r}}(t) - V_I)|. \tag{S1}$$

We use a Gaussian distance-dependent function to account for the subthreshold contributions from multiple nearby neurons, each at a certain location $\mathbf{r}' = (x', y')$:

$$LFP_{\text{space}}(|\mathbf{r} - \mathbf{r}'|) = \exp\left(\frac{-|\mathbf{r} - \mathbf{r}'|^2}{2\sigma^2_{LFP}}\right). \tag{S2}$$

We then apply a spatial convolution to the two components:

$$LFP(\mathbf{r}, t) = LFP_{\text{space}} * LFP_{\text{time}} = \int_{\mathbb{R}^2} LFP_{\text{space}}(|\mathbf{r} - \mathbf{r}'|)\ LFP_{\text{time}}(\mathbf{r}', t)\ d\mathbf{r}'. \tag{S3}$$

to give the LFP at $\mathbf{r} = (x, y)$ for time t.

For Equation S2, we use $\sigma_{LFP} = 125$ µm (3.125 gridpoints). This spatial decay value matches a recent experimental estimate of $\sigma_{LFP} = 100$ µm of the extent of stimulus-evoked LFP signals in primary visual cortex [7].

The stLFP measures the LFP caused by spikes originating from one location. To isolate this LFP contribution, we note that the spatiotemporal activity of our balanced, spatially extended network consists of propagating wave patterns with complex dynamics (see Results). Thus, we locate the trajectories of the patterns that occur around the RoI (i.e. within 10 gridpoints of the centre of the

RoI described by Eq. 4). Even in the absence of stimuli, patterns are still spontaneously generated in the network, so this a valid comparison between spontaneous and evoked cases. To do this, we use a flood-fill algorithm, as described in [8]. However, in addition to the two spatial dimensions of our network, the flood-fill algorithm also extends across the time dimension. For each gridpoint, we analyse the LFP only during those times when the centre-of-mass of an evoked pattern is nearby; here we consider "nearby" to be within a distance of 10 gridpoints but the results do not depend critically on this choice. We then calculate the amplitude of the LFP during the time period when the evoked pattern is nearby, which gives the stLFP for that gridpoint; if the evoked pattern is never nearby, the stLFP at that gridpoint is counted as 0. This procedure is applied to all gridpoints at which we measure the LFP, and repeated for all trials and patterns generated near the RoI to obtain an average.

Mean-matched Fano factor

The Fano factor (FF) measures the trial-to-trial variability of the spike count $n_{\mathbf{r}}(t-\frac{\Delta t}{2}, t+\frac{\Delta t}{2})$; here $t-\frac{\Delta t}{2}$ and $t+\frac{\Delta t}{2}$ indicate the start and end points, respectively, of the sliding time window, of size Δt, over which the spikes are counted. The FF of a neuron at $\mathbf{r}=(x,y)$ is then:

$$FF_{\mathbf{r}}(t) = \frac{\mathrm{var}[n_{\mathbf{r}}(t-\frac{\Delta t}{2}, t+\frac{\Delta t}{2})]}{\bar{n}_{\mathbf{r}}(t-\frac{\Delta t}{2}, t+\frac{\Delta t}{2})}, \tag{S4}$$

where $\bar{n}_{\mathbf{r}}(t-\frac{\Delta t}{2}, t+\frac{\Delta t}{2})$ and $var[n_{\mathbf{r}}(t-\frac{\Delta t}{2}, t+\frac{\Delta t}{2})]$ are the mean and variance, respectively, of the spike count across repeated trials with random initial conditions. Note that the FF of a homogeneous (with a constant rate of events) Poisson process is 1, whereas that of a periodic process is 0.

As in [2], we use mean-matching to calculate the FF; this ensures that a declining FF is not trivially related to rising rates (e.g. due to an increase in the size of the denominator in Eq. S4). The mean-matching method finds a common distribution of firing rates across all time steps, and ignores data points that do not fit this distribution [2]. To calculate this common distribution, we organise firing rates into histogram bins, which we choose to be of width 10 Hz. This gives matches for $\sim 31\%$ of all data points when the strongest stimulus is applied (1.2 nA). These matches are concentrated away from the centre of the RoI, where the firing rates are highest. On the other hand, firing rates around the centre of the RoI can be high enough for spike timing to be regularised by the refractory period, meaning that neural variability is quenched on both fast and slow time scales.

Skewness

To characterise the shape of the heavily-tailed distributions of membrane potentials, we calculate their skewness, as in [13]. For a neuron at position $\mathbf{r} = (x, y)$, the skewness is given by:

$$S(V_{\mathbf{r}}) = \frac{\frac{1}{L}\sum_{l=1}^{L}(V_{\mathbf{r}}(t^l) - <V_{\mathbf{r}}(t^l)>)^3}{(\frac{1}{L}\sum_{l=1}^{L}(V_{\mathbf{r}}(t^l) - <V_{\mathbf{r}}(t^l)>)^2)^{3/2}}, \tag{S5}$$

where $<V_{\mathbf{r}}(t^l)>$ is mean value of the voltage, L is the total number of samples in the time series, and $t^l = (l-1)\delta t$ with $\delta t = 1$ ms. A value of $S > 0$ indicates that the mean of the resultant distribution is greater than (to the right of) the median, whereas $S < 0$ indicates that the mean is less than (to the left of) the median; a Gaussian distribution has $S = 0$. If the skewness is positive/negative, this indicates that there is a heavy or a long-tail at large/small values. The skewness can also be calculated for distributions of synaptic inputs to neurons.

Firing rate model and analysis

To gain a theoretical understanding of response patterns in the spiking network, we consider a firing rate model that approximates the spiking model. Simplifying neural firing behaviour into binary states, i.e. the firing and resting states, has been widely used to analyse balanced networks [9, 14]. However, as the duration of the refractory period is comparable to or greater than that of the spiking period, it is more biologically plausible to simplify the neural firing process into three states, i.e. the firing, resting and refractory states [3]. This simplification replaces the physiological variable of membrane potential, possessed by LIF neurons, with one of these three states. Following [12], we assume random spikes, approximate the synaptic input by a Gaussian distribution, and replace the discrete lattice with a continuum of spatial positions so that we obtain:

$$\begin{cases} \frac{\partial f}{\partial t} &= -f + (1 - f - h)H(s - \kappa), \\ \frac{\partial h}{\partial t} &= -ph + f, \end{cases} \tag{S6}$$

where $f(x, y, t)$ and $h(x, y, t)$ represent the fraction of neurons that are in the firing and refractory states, respectively; resting neurons $j(x, y, t)$ are factored out according to $j = 1 - f - h$, i.e. the condition that all neurons must be in one of the three states. In Equation S6, $\kappa = 1$ is the firing threshold; $p = 0.30$ is a control parameter, which is chosen to ensure that the spontaneous dynamics of the rate model are the same as that of the spiking circuit model; and $H(\cdot)$ is a Heaviside function.

Input $s(x,y,t)$ is:

$$s(x,y,t) = u(x,y,t) + g(x,y,t), \tag{S7}$$

where $u(x,y,t) = w * f$ is the synaptic input with $*$ indicating a spatial convolution, w is the neural coupling strength, and $g(x,y,t)$ is a localised external stimulus, similar to that applied in the spiking circuit model.

In the spiking circuit model, the strength of inhibitory coupling is relatively larger than that of excitatory coupling at longer ranges. To approximate such a distance-dependent coupling rule, we use Bessel functions due to their analytic tractability [4]:

$$w(r) = W_E w_K(r/\sigma_E) - W_I w_K(r/\sigma_I) \tag{S8}$$

where $r = \sqrt{x^2+y^2}$ is the Euclidean distance, and $w_K(r) = \frac{2}{3\pi}[K_0(r) - K_0(2r)]$ where K_ν is the modified Bessel function of the second kind of order ν; and the subscripts E and I denote excitatory and inhibitory coupling, respectively. The function $w_K(r)$ approximates the exponential function $\frac{1}{2\pi}e^{-r}$ and it is normalised such that $\int_{\mathbb{R}^2} w_K(r)d\boldsymbol{r} = 1$. To ensure that the firing rate model has the same ratio of balance of excitation and inhibition as in the spiking circuit model, we first measure the excitatory synaptic currents $C_E(t) = g_E(t)[V(t) - V_E]$ and inhibitory synaptic currents $C_I(t) = g_I(t)[V(t) - V_I]$ of each neuron in the spiking circuit model. We then calculate the ratio $\beta = <\bar{C_E}> / <\bar{C_I}>$ where $<\cdot>$ indicates averaging over time and the bar indicates averaging across neurons. The connectivity parameters of the firing rate model are then chosen to guarantee that the excitatory and inhibitory areas of $w(r)$ have the same ratio as β; the resultant parameters are: $W_E = 159.4$, $W_I = 73.7$, $\sigma_E = 1.87$ and $\sigma_I = 3.24$. Note that while we use uniform inhibition in our model (up to a distance d_{max}), the use of a decay term here enables the standard neural field analysis, as used in Qi et al. [12], to be applied to the center-surround connectivity of the spiking model. For the firing rate model, a sudden decay in inhibition, as found in our spiking model, yields similar qualitative results [5, 6]

To study the response properties of the firing rate model, we add a localised input centred at the RoI, as in the spiking circuit model. This is given by the second term in Equation S7, which is:

$$g(x,y,t) = W_S \exp\left[-\frac{(\sqrt{(x-\bar{x})^2 + (x-\bar{y})^2}}{\sigma_S}\right]. \tag{S9}$$

This describes a stimulus centred at $(\bar{x}, \bar{y})$ with a Gaussian profile of amplitude W_S that spatially decays according to $\sigma_S = 1.61$.

For a localised patchy pattern with radius a, the firing rate model satisfies $s(r) < \kappa$ for $r > a$ and $s(r) > \kappa$ for $r < a$. In this case, the firing rate model has a steady-state solution in the form of a uniform disk $\bar{f}(r) = \frac{p}{1+2p}$ and $\bar{h}(r) = \frac{1}{1+2p}$ for $r < a$ and $\bar{f}(r) = \bar{h}(r) = 0$ for $r > a$ [12]. The corresponding synaptic input is:

$$\bar{u}(r) = w * \bar{f} = \frac{p}{1+2p}\mathcal{I}(r), \tag{S10}$$

$$\mathcal{I}(r) = \int_0^{2\pi}\int_0^a w(|\boldsymbol{r} - \boldsymbol{r}'|)r'dr'd\theta'. \tag{S11}$$

By imposing the boundary condition $\bar{u}(a)+g(a) = \kappa$, we obtain an existence condition for a radially symmetric bump:

$$g(a) = \frac{(1+2p)\kappa - p\mathcal{I}(a)}{1+2p}. \tag{S12}$$

The dependence of the radius a may then be calculated by using a root finding routine. This yields the two solutions with small and large radius, as depicted in Figure 1f.

Next we calculate whether the conditions depicted in Figure 1f are stable to perturbation. The general eigenvalue problem is the same as that derived in [12]

$$\begin{bmatrix} \lambda+1 & 1 & 1 \\ 0 & \lambda+2 & 1 \\ 0 & -1 & \lambda+p \end{bmatrix}\begin{bmatrix} \mu(\boldsymbol{r}) \\ \Phi(\boldsymbol{r}) \\ \Psi(\boldsymbol{r}) \end{bmatrix} = \begin{bmatrix} \mathcal{I}_+ + \frac{p}{1+2p}\mathcal{I}_- \\ \mathcal{I}_+ \\ 0 \end{bmatrix} \tag{S13}$$

where, in our case,

$$\mathcal{I}_\pm = \frac{a}{|\bar{u}'(a) + g'(a)|}\int_{\mathcal{A}_\pm} \mu(a, \theta', t)w(|\boldsymbol{r} - \boldsymbol{a}')d\theta' \tag{S14}$$

and $\mathcal{A}_\pm$ denotes the set of points on the localised patch boundary with positive or negative perturbations, respectively.

Using the approach of [12], exact analytical expressions can be given for both the radially symmetric contraction and the radially symmetric expansion, the only difference being that $u(a)$ is replaced by $u(a) + g(a)$. In this case, we find that for radially symmetric contraction, the solution with smaller radius is always unstable and the solution with larger radius is undetermined (i.e. it has nonzero imaginary parts) for the range of values W_S that we model. Likewise, for radially symmetric expansion, we find that the solution with the smaller radius is always unstable and the solution with the larger radius is always stable for the range of values W_S that we model.

Finally, we consider general cases with radially asymmetric perturbations. There is no systematic

way to obtain analytical solutions to the eigenvalue problem (Eq. S13), so we apply the following numerical scheme, as introduced by [1] and outlined by [12]. First, we discretise the spatial coordinate as $\theta_j = 2\pi\frac{j}{N}$, $j = 0, 1, \ldots, N-1$, and consider perturbations such that $\mathcal{A}_\pm$ is the union of $n \geq 1$ disconnected, evenly distributed, and equally sized subregions with $\mu(a, \theta_i) \lessgtr 0$. Then, to fully characterise the set of possible eigenmodes for each n, the size of l of each subregion of $\mathcal{A}_\pm$ is taken to be anywhere from 1 to $N/n - 1$. Let

$$w_{jk} = w\left(2a\,\sin\frac{\theta_j - \theta_k}{2}\right) \tag{S15}$$

and approximate the integral of Equation S14 using:

$$\mathcal{I}_\pm(a, \theta_j) \approx \frac{a}{|\bar{u}'(a) + g'(a)|}\frac{2\pi}{N}\sum_{\mathcal{A}_\pm}\mu(a, \theta_k)w_{jk}. \tag{S16}$$

Applying this approximation to Equation S13, we obtain a matrix eigenvalue problem, conveniently written in block matrix form:

$$\begin{bmatrix} W^+ + \frac{p}{1+2p}W^- - I & -I & -I \\ W^+ & -2I & -I \\ 0 & I & -pI \end{bmatrix}\begin{bmatrix} \mu(\boldsymbol{r}) \\ \Phi(\boldsymbol{r}) \\ \Psi(\boldsymbol{r}) \end{bmatrix} = \lambda\begin{bmatrix} \mu(\boldsymbol{r}) \\ \Phi(\boldsymbol{r}) \\ \Psi(\boldsymbol{r}) \end{bmatrix} \tag{S17}$$

where I is the $N \times N$ identity matrix. The matrix $W^\pm$ is an $N \times N$ matrix whose entries are given by:

$$W^+_{jk} = \frac{a}{|\bar{u}'(a) + g'(a)|}\frac{2\pi}{N}w_{jk}\mathbf{1}^\pm_k \tag{S18}$$

where the indicator function $\mathbf{1}^\pm_k$ is equal to 1 if $\theta_k \epsilon \mathcal{A}_\pm$ and 0 otherwise. For each mode n, we solve the eigenvalue problem of Equation S17 numerically for every l and find the corresponding eigenfunction $\mu(a, \theta)$. A valid solution to the eigenfunction is found if either $\mu(a, \theta)$ and $-\mu(a, \theta)$ satisfies the initial assumption about the signs of the perturbations. Because we have already shown that the smaller radius solution is unstable to symmetric perturbations, we only apply this analysis to the larger radius solution. This is because, while the larger radius solution is stable for symmetric perturbations, it may not be stable for higher-order, asymmetric perturbations, namely shift perturbations of order $n = 1$.

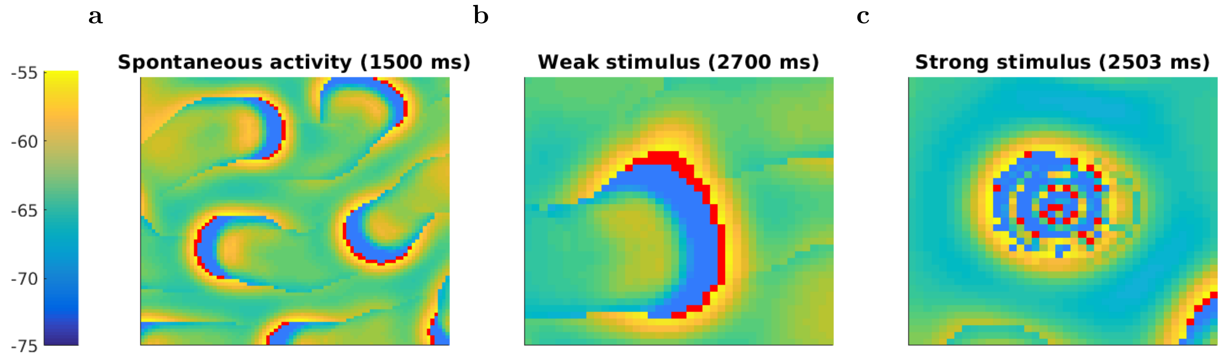

Supplementary Materials, Figure S1. Patterns emerging from a network with distance-dependent inhibition. This figure demonstrates that the qualitative behaviour of wave patterns does not depend on the uniform inhibitory strength, and is similar to Fig. 1(a-c). In this case, we use the same parameters as detailed in Methods except that inhibition in Eq. 3 is given by $W_I \exp\left(\frac{-|\mathbf{r}-\mathbf{r}'|^2}{2\sigma_I^2}\right)$ where $W_I = 5.0 \times 10^{-3}$ nS and $\sigma_I \approx 380$ µm. Colours in the images represent membrane potential values between -75 mV (blue) and spike threshold $V_{th} = -55$ mV (yellow), as indicated by the colour bar, with red pixels indicating neurons that have fired within the last millisecond. Each image is of a network of size 300×300 neurons, but a smaller range is shown for clarity, namely an 81×81 subsection in (a) and a 41×41 subsection centred around the Region of Input (RoI) in (b) and (c). (a) Snapshot of the spatiotemporal patterns emerging from the balanced, spatially extended network during the spontaneous activity. These patterns mostly take the form of multiple, crescent-shaped propagating waves. (b) Snapshot of a propagating wave evoked by a weak stimulus (0.2 nA) within the RoI. (c) Snapshot of a localised patchy pattern evoked by a strong stimulus (1.2 nA) within the RoI.

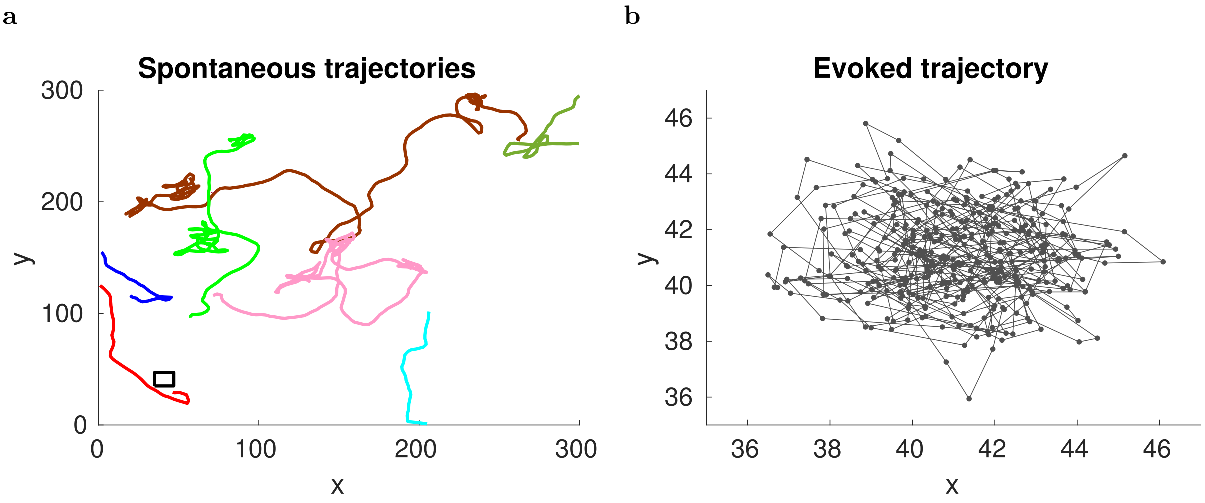

Supplementary Materials, Figure S2. Typical pattern trajectories. Both x and y axis are in units of gridpoints. (a) A sample of the trajectories of waves occurring during spontaneous activity in the model. Both the initiation sites and propagation paths of these waves appear to be random. The trajectories are terminated as a result of interactions with other waves. (b) Trajectory of a pattern evoked and trapped by a strong, localised stimulus. The pattern tends to "wander" around a particular area, and covers a smaller region than the spontaneous waves; note the smaller 12×12 scale of this figure, also depicted by the black rectangle in (a).

References

[1] P C Bressloff and Z P Kilpatrick. Two-dimensional bumps in piecewise smooth neural fields with synaptic depression. *SIAM J Appl Math*, 71(2):379–408, 2011.

[2] Mark M Churchland, Byron M Yu, John P Cunningham, Leo P Sugrue, Marlene R Cohen, Greg S Corrado, William T Newsome, Andrew M Clark, Paymon Hosseini, Benjamin B Scott, David C Bradley, Matthew A Smith, Adam Kohn, J Anthony Movshon, Katherine M Armstrong, Tirin Moore, Steve W Chang, L H Snyder, S G Lisberger, Nicholas J Priebe, Ian M Finn, David Ferster, Steven I Ryu, Gopal Santhanam, Maneesh Sahani, and Krishna V Shenoy. Stimulus onset quenches neural variability: a widespread cortical phenomenon. *Nat Neurosci*, 13:369–378, 2010.

[3] J D Cowan. Stochastic neurodynamics. In R P Lippman, J E Moody, and D S Touretzsky, editors, *Advanced in neural information processing systems*, volume 3, pages 62–65. Moogan Kaufmann Publishers, San Mateo, CA, 1991.

[4] S E Folias and P C Bressloff. Breathers in two-dimensional neural media. *Phys Rev Lett*, 95:208107, 2005.

[5] Pulin Gong, S. T. C. Loi, P. A. Robinson, and C. Y. J. Yang. Spatiotemporal pattern formation in two-dimensional neural circuits: roles of refractoriness and noise. *Biol Cybern*, 107(1):1–13, 2013.

[6] Pulin Gong and Peter A. Robinson. Dynamic pattern formation and collisions in networks of excitable elements. *Phys Rev E*, 85(5), 2012.

[7] S Katzner, I Nauhaus, A Benucci, V Bonin, D L Ringach, and M Carandini. Local origin of field potentials in visual cortex. *Neuron*, 61:35–41, 2009.

[8] A. Keane and P. Gong. Propagating waves can explain irregular neural dynamics. *J Neurosci*, 35:1591–1605, 2015.

[9] Ashok Litwin-Kumar and Brent Doiron. Slow dynamics and high variability in balanced cortical networks with clustered connections. *Nat Neurosci*, 15(11):1498–1505, 2012.

[10] Alberto Mazzoni, Henrik Lindén, Hermann Cuntz, Anders Lansner, Stefano Panzeri, and

Gaute T Einevoll. Computing the local field potential (LFP) from integrate-and-fire network models. *PLoS Comput Biol*, 11(12):e1004584, 2015.

[11] Ian Nauhaus, Laura Busse, Matteo Carandini, and Dario L Ringach. Stimulus contrast modulates functional connectivity in visual cortex. *Nat Neurosci*, 12(1):70–76, 2009.

[12] Yang Qi and Pulin Gong. Dynamic patterns in a two-dimensional neural field with refractoriness. *Phys Rev E*, 92:022702, 2015.

[13] A Y Tan, Y Chan, B Scholl, E Seidemann, and N J Priebe. Sensory stimulation shifts visual cortex from synchronous to asynchronous states. *Nature*, 509:226–229, 2014.

[14] C van Vreeswijk and H Sompolinsky. Chaotic balanced state in a model of cortical circuits. *Neural Comput*, 10(6):1321–1371, 1998.
